# Fruit and Vegetable Knowledge and Intake within an Australian Population: The AusDiab Study

**DOI:** 10.3390/nu12123628

**Published:** 2020-11-25

**Authors:** Caroline R. Hill, Lauren C. Blekkenhorst, Simone Radavelli-Bagatini, Marc Sim, Richard J. Woodman, Amanda Devine, Jonathan E. Shaw, Jonathan M. Hodgson, Robin M. Daly, Joshua R. Lewis

**Affiliations:** 1 Institute for Nutrition Research, School of Medical and Health Sciences, Edith Cowan University, Perth, WA 6000, Australia; l.blekkenhorst@ecu.edu.au (L.C.B.); s.radavellibagatini@ecu.edu.au (S.R.-B.); marc.sim@ecu.edu.au (M.S.); a.devine@ecu.edu.au (A.D.); jonathan.hodgson@ecu.edu.au (J.M.H.); joshua.lewis@ecu.edu.au (J.R.L.); 2 Medical School, The University of Western Australia, Nedlands, WA 6009, Australia; 3 Flinders Centre for Epidemiology and Biostatistics, Flinders University, Bedford Park, SA 5042, Australia; richard.woodman@flinders.edu.au; 4 Clinical Diabetes and Epidemiology, Baker Heart and Diabetes Institute, Melbourne, VIC 3004, Australia; jonathan.shaw@baker.edu.au; 5 School of Public Health and Preventive Medicine, Monash University, Clayton, VIC 3800, Australia; 6 Institute for Physical Activity and Nutrition, School of Exercise and Nutrition Science, Deakin University, Burwood, VIC 3125, Australia; robin.daly@deakin.edu.au; 7 Centre for Kidney Research, Children’s Hospital at Westmead School of Public Health, Sydney Medical School, The University of Sydney, Sydney, NSW 2006, Australia

**Keywords:** health promotion, literacy, eating, diet, survey, questionnaire, fruit, vegetables

## Abstract

Understanding the relationship between fruit and vegetable knowledge (FVK) and fruit and vegetable intake (FVI) is an important consideration for improved public health and successful targeting of health promotion messaging. The aim of this study was to investigate the association between FVK and FVI in Australian adults and to identify subgroups most at risk of poor knowledge. Using data from the Australian Diabetes, Obesity, and Lifestyle Study (AusDiab), we investigated associations between FVK and FVI, as well as demographic and lifestyle factors. Baseline FVK was measured using two self-reported questions. FVI was assessed using a validated, self-reported, food frequency questionnaire in 1999/00 (baseline), 2004/05, and 2011/12. Amongst the 8966 participants assessed at baseline, 24.1% had adequate, 73.0% had insufficient, and 2.9% had poor FVK. Using linear regression, those with insufficient or poor FVK reported significantly lower FVI (grams/day) compared to those with adequate FVK: baseline (coefficient (95%CI)): −67.1 (−80.0, −54.3) and −124.0 (−142.9, −105.1), respectively, whilst, at 12 years, the differences were −42.5 (−54.6, −30.5) and −94.6 (−133.8, −55.5) grams/day, respectively (all *p* < 0.001). Poor FVK was more likely to be reported in males, older individuals (>65 years), socio-economically disadvantaged, smokers, and those with insufficient physical activity/sedentary behavior. We demonstrate that having adequate knowledge of FVI, defined as knowing to consume fruit and vegetables several times a day for a well-balanced diet, is strongly associated with FVI, with several demographic and lifestyle factors predicting FVK. Health promotion messages aimed at increasing FVK should target these subgroups for maximal effect.

## 1. Introduction

Lifestyle-related chronic diseases, such as diabetes and cardiovascular disease (CVD), continue to maintain their status as the leading cause of death worldwide [1]. Whilst ‘optimal’ fruit and vegetables intakes (FVI) is yet to be established, a large meta-analysis across 95 studies suggests that as many as 5.6 and 7.9 million deaths in 2013 were due to fruit and vegetables intakes (FVI) below 500 and 800 g/day, respectively [2]. Furthermore, in many countries, the vast majority of adults fail to even achieve the daily fruit and vegetable recommendations of ≥400 g, as encouraged by the World Health Organization [3,4,5,6].

Specific phytochemicals within fruits and vegetables have shown multiple benefits for lowering chronic disease risk [7,8,9], such as organosulfur compounds in cruciferous and alliums, nitrate and vitamin K in green leafy vegetables, and saponins and phenolic compounds in legumes [10]. Carotenoids and phenolic compounds, such as flavonoids, found in many fresh fruits, also show health promoting benefits [8,9]. Higher fruit and vegetable intake overall is associated with reduced risk of cardiovascular disease [11,12,13,14], cancer [2], and all-cause mortality [2,13,15].

Considering these extensive benefits, understanding why habitual FVI remain poor is of major interest for public health professionals. There have been numerous barriers identified that influence FVI, including time, taste preferences, self-efficacy, inadequate cooking skills, social support, motivation, and willpower [16,17,18,19,20]. Those with lower diet quality and reduced FVI are also more likely to come from lower socio-economic backgrounds [21,22,23], more likely to consume higher discretionary foods [23], and more likely to have lower health literacy and nutrition knowledge [24,25,26].

Nutrition knowledge has been associated with improved dietary behaviors, including increased FVI [27,28,29]. Several theoretical frameworks seek to understand and improve dietary behaviors, with knowledge widely considered an integral part [30]. For example, the Theory of Planned Behavior (TPB) [31] and Social Cognitive Theory (SCT) [32] are common in public health in order to explain, predict, and change health behaviors. Nutrition-specific knowledge remains a core component of nutrition literacy, supporting the progress toward healthy choices [33]. Despite this, there remains multiple components of knowledge; knowing nutrition facts (i.e., declarative knowledge) does not necessarily always translate into improved dietary behaviors (i.e., procedural knowledge, such as how to prepare food) [30]. For example, Wallace et al. [34] describe using SCT among the elderly with dementia to raise nutrition literacy resulting in increased variety of vegetables and improved dietary behaviors. However, incorporating TPB into a randomized trial specifically to raise FVI among young adults found little correlation between intent and behavior (i.e., FVI) in the intervention group [35], indicating the complexity of the knowledge-behavior relationship.

Nevertheless, higher nutrition knowledge of fruit and vegetables and how often they should be consumed have been identified as factors influencing FVI [28]. Yet, high quality studies that explore subgroups, particularly those for whom FVI appears lowest, such as socio-economically disadvantaged, males, and lower educated people, are needed to direct further public health research. With this in mind, there are limited studies investigating the association between fruit and vegetable knowledge and actual intake, both within the global context and from an Australian perspective. Even less has been reported on the impact of fruit and vegetable knowledge on medium and long-term FVI, presenting a gap needing to be explored within this field.

Therefore, this study aimed to explore whether knowing to consume fruit and vegetables several times a day for a well-balanced diet is associated with a higher FVI among Australian adults aged ≥25 years. We also sought to determine which subgroups within this cohort have lower fruit and vegetable knowledge, as well as which subgroups have lower FVI over 12 years of follow-up. By identifying those most at risk, additional and targeted public health messages can be implemented to increase FVI within these subgroups. Determining if such declarative knowledge (i.e., knowing how often to consume fruit and vegetables) impacts FVI will be an important contribution in the design of future public health messages. To our knowledge, we are the first to explore these associations over the medium to long-term across such a large cohort within the Australian population.

## 2. Materials and Methods

### 2.1. Data Collection

This cross-sectional and prospective study was conducted using data from the Australian Diabetes, Obesity, and Lifestyle (AusDiab) study. As reported previously [36], AusDiab is a large population-based study involving a randomly selected sample of 11,247 Australian adults aged ≥25 years. The sample was collected across 42 randomly selected census districts across the Northern Territory and Australia’s six states. Each of these areas were selected using a selection probability proportional to the size of their population aged ≥25 years. The original aim of the study was to explore the prevalence, risk factors, trends, knowledge, attitudes, and use of health services associated with diabetes mellitus and obesity within the Australian adult population. Interviews, anthropometrics, and blood measurements were collected on demographics, socio-economic status (SES), health and disease conditions, dietary intake, physical activity, and lifestyle habits, as well as attitudes and knowledge of a range of health-related areas. The original data collection occurred during May 1999 through to December 2000, with follow-ups in 2004/05 and 2011/12. The full study details and response rates are described elsewhere [36]. For this current study, only certain components of the original data collection were extracted for quantitative analysis.

Participant Selection

Participants were excluded if they were either pregnant or did not know they were pregnant (*n* = 89), or if they did not complete either the Food Frequency Questionnaire (FFQ) (*n* = 203) or the knowledge questionnaire (*n* = 1241). Those who reported consuming an implausible energy intake (defined as either <3.3 or >17.5 MJ/day in men and <2.5 or >14.5 MJ/day in women) were also excluded from analysis (*n* = 328). Those with missing data for covariates were excluded (*n* = 420). The final participant number for the cross-sectional analysis was 8966.

**Figure 1:**
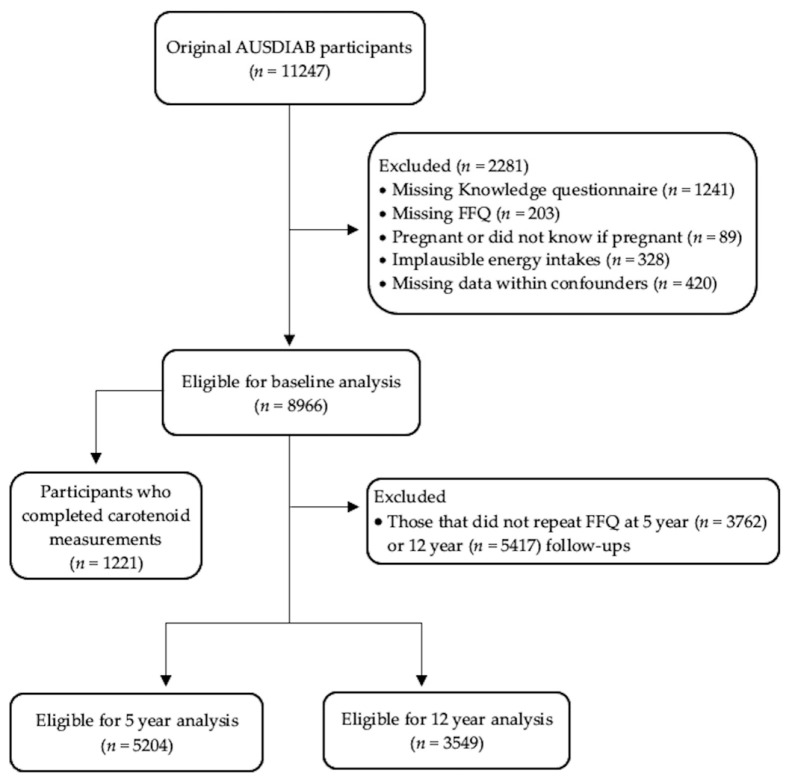
Participant flow diagram.

For the prospective component of this study, FVI from repeat FFQs was analyzed from 5204 participants after 5 years (collected during 2004/2005) and from 3549 participants at 12 years (collected during 2011/2012). Those participants not included had either dropped out or failed to complete the necessary FFQ (42% in 2004/05 and 60% in 2011/12, respectively). A participant flow diagram is illustrated in Figure 1.

### 2.2. Variables

#### 2.2.1. Fruit and Vegetable Knowledge

To determine fruit and vegetable knowledge of adequate intake, a score was calculated for each participant using raw data taken from the ‘AusDiab Health Knowledge, Attitudes, and Practice Questionnaire’ [37]. This questionnaire was administered at baseline by a trained interviewer. Knowledge of fruit and vegetable consumption was acquired using the following two questions; ‘For a well-balanced diet, how often should adults eat FRUIT?’ and ‘For a well-balanced diet, how often should adults eat VEGETABLES?’ (questions 7 and 9). A weighting of 25 was applied to those that responded ‘several times a day’, 20 applied to ‘daily’, 15 to ‘every second day’, 10 to ‘twice a week’, 5 to ‘weekly or less often’, and a weighting of 0 to those who answered ‘don’t know’, for each question. This resulted in a five-point interval scale for fruit and vegetable knowledge. After assigning these weighting scores to participant responses, the authors of this study further created a self-derived ‘fruit knowledge score’ and a ‘vegetable knowledge score’, as well as a combined ‘fruit and vegetable knowledge score’, for each individual, with a maximum possible score of 25, 25, and 50, respectively. A score of <20 for either fruit or vegetables alone represented poor knowledge, and a score of 20 was insufficient, whilst 25 indicated adequate knowledge. Meanwhile, a score of <40 for fruit and vegetables combined represented poor knowledge, 40–45 indicated insufficient knowledge, and a maximum score of 50 indicated adequate fruit and vegetable knowledge levels. That is, a participant had to be adequate in both fruit knowledge and vegetable knowledge to be assigned the adequate score for total fruit and vegetable knowledge. As the knowledge questionnaire was administered once (i.e., at the commencement of the study), all fruit knowledge, vegetable knowledge and combined fruit and vegetable knowledge scores reflect a participant’s knowledge only at baseline.

#### 2.2.2. Fruit and Vegetable Intake

Dietary intake of fruit and vegetables was self-reported using the validated Anti-Cancer Council of Victoria Food Frequency Questionnaire, developed by the Cancer Council of Victoria [37,38,39]. Participants estimated how many pieces of fruit and how many different vegetables they typically ate each day. Responses were then verified using photographs to estimate serving sizes. We calculated total fruit intake, excluding fruit juice and tinned fruits due to their potential for high added sugar content, whilst total vegetable intake excluded hot chips as these are not recommended as part of a healthy eating pattern [40]. Table results display FVI as a continuous measurement in grams/day. The daily intake in serves was also calculated by dividing the total intake in grams by the recommended serving sizes as defined by the Australian Dietary Guidelines [40]. Therefore, one serving of fruit was 150 g/day, and one serving of vegetables was 75 g/day [40]. To calculate the proportion of participants meeting FVI recommendations, males were stratified into ≤50 years, 51–70 years, and >70 years, due to their differing serving size requirements [40].

#### 2.2.3. Serum Carotenoids

Serum carotenoid levels (mmol/L) were measured at baseline in a subsample of participants (*n* = 1598), randomly selected from six areas in Queensland, Australia [41]. The carotenoids included were α-carotene, β-carotene, β-cryptoxanthin, lutein/zeaxanthin, and lycopene, with each individually assayed using high performance liquid chromatography under procedure guidelines described [42]. A total carotenoid score was derived based on the sum for each of the aforementioned carotenoids, and, after our exclusion criteria was applied for this current study, a total of 1221 participants were included for analysis of carotenoid levels.

### 2.3. Baseline Demographics and Assessments

The following variables were collected at baseline and explored as confounders and predictors to intake: age (date of birth), sex (male/female), body mass index (BMI) (kg/m^2^), energy intake (MJ/day), relationship status (married, de facto, separated, divorced, widowed, or never married), physical activity level (sedentary as nil, insufficient <150 min per week, or sufficient >150 min per week), educational attainment (never to some high school or completed university or equivalent), smoking status (current, former-smoker, or never smoked), SEIFA (Socio-Economic Indexes for Areas) disadvantage score, self-reported history of CVD (yes/no), and the presence of Diabetes Mellitus (known Diabetes Mellitus, impaired fasting glucose, impaired glucose tolerance, new Diabetes Mellitus, or normal glucose levels). Full collection and measurement details have been described previously [36]. Briefly, anthropometrics for BMI were measured as described [43] and physical activity as per the Active Australia Survey Questionnaire [44]. For this particular study, we grouped participants based upon age (25–45 years, 45–65 years, >65 years) to represent young, middle-aged, and older-adult Australians. BMI groups were created based on universally accepted classifications [45]. Participants’ SEIFA disadvantage scores were also grouped into quartiles according to cut-points predefined by 2001 Census data [46].

### 2.4. Statistical Analysis

Analyses were performed using STATA statistical software (version 15 StataCorp, College Station, TX, USA), with all cross-sectional analyses using the survey command to apply the necessary weighting for selection bias as a result of either under or over-sampling. The weighting applied was based upon 1998 Australian Census data for sex and age distribution. Prior to commencing analysis, the normality and distribution of variables were assessed to ensure all assumptions were met. Descriptive statistics are presented as weighted means and standard deviations, or weighted observations and percentages, where appropriate. These include baseline descriptives of participants, and their characteristics according to baseline knowledge scores, for both total fruit and vegetable knowledge, and fruit knowledge and vegetable knowledge, separately. All statistical outcomes are provided with a confidence limitation set at 95%.

Linear regression was used to assess the relationship between baseline fruit and vegetable knowledge score categories (adequate, insufficient, and poor) and FVI at baseline, 5 years, and 12 years of follow-up, before and after adjustment for demographic and lifestyle factors. Adequate fruit and vegetable knowledge was used as the referent category. Model 1 was unadjusted, whilst model 2 included age, sex, education level, SEIFA disadvantage, energy intake, marital status, physical activity level, smoking status, BMI, presence of CVD, and diabetes status. Linear regression also assessed the association of baseline knowledge on intake, for both fruit and vegetables separately, as well as the association of the aforementioned demographic and lifestyle factors, upon intake. The positive correlation between FVI and total serum carotenoids, a marker of FVI, has been previously reported in a subgroup of this cohort [41]. We also explored the correlation between total carotenoids and fruit and vegetable knowledge categories in the subsample for whom carotenoids were measured. For this, we applied a square root log transformation on our continuous total carotenoid variable to normalize its distribution prior to applying survey command.

### 2.5. Ethical Approval

The AusDiab study was approved by the International Diabetes Institute Ethics Committee (Melbourne, Australia) and by The Human Research Ethics Committee of the Alfred Hospital (Melbourne, Australia).

## 3. Results

### 3.1. Demographic Characteristics

A total of 8966 individuals (49.8% men) were included in our final cross-sectional analysis (Table 1). The mean weighted age was 47.9 years (*SD* = 15.0) and ranged from 25–91 years, whilst the mean weighted BMI was 26.6 kg/m^2^ (*SD* = 4.8). Those with adequate baseline fruit and vegetable knowledge scores were more likely to be female, younger, have completed university or equivalent, be a non-smoker, more socio-economically advantaged, physically active, married, and consume lower overall energy intake. Those with either CVD or diabetes were more likely to have the lowest (i.e., poor) baseline fruit and vegetable knowledge scores. There was no observed difference in a participant’s knowledge score according to BMI status.

When we explored baseline fruit and vegetable knowledge separately, those with adequate fruit knowledge were more likely to be female, consume lower overall energy intake, have completed university, be less socio-economically disadvantaged, not have diabetes, and not smoke (Supplementary Table S1). Fruit knowledge scores had no observed differences in reported physical activity level, marital status, BMI, or age group, nor in the presence of CVD. Those having adequate vegetable knowledge, however, were more likely to be female, younger, report sufficient physical activity levels, and be married. Adequate vegetable knowledge was also more likely to be observed in those who were more educated, less socio-economically disadvantaged, non-smokers, and to not have either CVD or diabetes. Neither BMI group nor overall energy intake appeared to have any observed differences in a participant’s vegetable knowledge score.

### 3.2. Fruit and Vegetable Knowledge and Intake

Participants with adequate fruit and vegetable knowledge at baseline were found to also have greater FVI overall at baseline, in both our unadjusted and adjusted models (see Table 2).

In our adjusted model, we found that increasing age (both 45–65 year and >65 year age groups), being female, consuming greater energy intake, engaging in >150 min of physical activity per week, being a known diabetic, and either a former or non-smoker was significantly and positively associated with higher FVI (see Table 3).

Variables associated with FVI separately were also explored at baseline (Supplementary Table S2). Whilst differences occurred when observing FVI separately, of significance was that having completed university or equivalent was significantly associated with less vegetable intake (β = −11.9, *p* = <0.001), as was being widowed (β = −13.6, *p* = 0.008) over those who were married. Those not reporting CVD consumed less vegetable intake (β = −12.9, *p* < 0.001). Those in a de facto relationship consumed significantly lower fruit (β = −19.9, *p* = 0.026) over their married counterparts, and those most socio-economically disadvantaged consumed higher fruit (β = 9.6, *p* = 0.048).

### 3.3. Fruit and Vegetable Knowledge and Intake over 12 years

Linear regression analysis found that insufficient and poor knowledge at baseline was significantly associated with lower FVI at both 5 and 12 years, in both our unadjusted and adjusted models (Table 4).

### 3.4. Fruit and Vegetable Knowledge and Serum Carotenoids

We found a weakly positive, although significant, association between total serum carotenoids and baseline fruit and vegetable knowledge (r = 0.14, *p* = 0.006). This correlation with carotenoids was similar for both fruit knowledge (r = 0.14, *p* = 0.021) and vegetable knowledge (r = 0.12, *p* = 0.011) separately.

## 4. Discussion

In our study, we observed that only a quarter of subjects knew to consume fruit and vegetables several times a day, for a well-balanced diet. Adequate knowledge was positively associated with self-reported FVI and total serum carotenoids, a biomarker of FVI. The positive association of adequate knowledge on overall intake was evident and remained even after controlling for many demographic and lifestyle factors. Our results support the continuation of public health messages to promote fruit and vegetable knowledge in order to raise habitual intakes across the Australian population. Specifically, we found particular attention may be warranted for males, older Australians (>65 years), those most socio-economically disadvantaged, current smokers and those not engaging in sufficient physical activity as these attributes were each associated with lower knowledge scores. Furthermore, novel methods to engage the entire population to improve knowledge and increase FVI should be a high priority, particularly for vegetables, with reported intakes across our entire cohort highly inadequate. This clearly illustrates that, whilst we observed a higher FVI in those reporting higher FVK, this by no means implies that those with ‘adequate’ FVK, as assessed by the AusDiab questionnaire, cannot still potentially improve other aspects of their FVK. Unique to this study was our ability to assess weighted mean FVI at three separate timepoints over 12 years, as well as to demonstrate that the association with baseline knowledge remained, but also weakened, over time.

### 4.1. The Association between Fruit and Vegetable Knowledge and Intake

Having higher knowledge, for both fruit and vegetables separately, or fruit and vegetables combined, was associated with higher FVI. For those with adequate knowledge, there was an associated higher (mean 124.0 g/day) FVI in comparison to those with poor knowledge. At 5 and 12 years, this association was slightly attenuated (mean difference 122.2 g/day and 94.6 g/day, respectively) although the positive association remained in both unadjusted and adjusted models. Whilst other observational studies have also identified a positive association between knowledge and intake [17,27,47,48,49,50,51,52], this study appears to be the first to provide evidence of this relationship over the long term. Previous intervention studies (typically involving a pre-post knowledge or an education component), for the most part, have found similar positive results with knowledge increasing FVI by ~0.1–1.8 cups per day, within a three-month duration [53,54,55,56,57,58]. Meanwhile, a systematic review and meta-analysis by Lara et al. [59] found that dietary education increased FVI by 86 g per day after 4 to 12 months, which was maintained (87 g per day) after 13–58 months. Nonetheless, not all education programs have successfully resulted in increases in FVI over time [60,61]. A study in Western Australia (*n* = 2854) found that, despite increased awareness of the “Go for 2&5” fruit and vegetable campaign, FVI actually decreased over a nine-year period [62]. Participants perceived that their intake of fruits and vegetables was already adequate (34.5% and 59.3%, respectively), reporting that insufficient time and preparatory efforts required as the main difficulties to increasing consumption. These findings support the existence of additional barriers toward FVI, particularly for vegetables, that remain unanswered [17,20].

Previously identified barriers to FVI include neophobias, habits, taste preferences, religion, time-constraints, convenience, perceived cost, insufficient self-efficacy, and social support [16,20,63]. Moreover, the overall reduced consumption of vegetables, along with the lower influence of knowledge upon intake, when compared to fruit, suggests greater barriers to vegetable intake. This has been reported by others [16,61,64] inferring that public health messages targeting vegetables separately are warranted. Multiple barriers have been identified to increasing vegetable intake, including one’s lack of cooking and preparatory skills, plus the additional time requirements for cooking [65]. Furthermore, studies have also found that often people are unaware their consumption is low, particularly for vegetables [16]. Despite widespread awareness of Western Australia’s 5&2 campaign, participants assumed this vegetable recommendation was ‘aspirational’, unachievable, and unnecessary for any greater health benefit [62,63,66]. Often, knowledge is the first step in behavior change [30], and, as such, greater efforts to target fruit and vegetable messages across the entire population are needed and confirmed by these findings.

### 4.2. Targeting Those at Risk

In line with previous studies, we found that fruit and vegetable knowledge was highest among females [27,48,67,68,69], the younger-to-middle aged [28,48,70], those more educated [51,52,68,69,70], married [27,68], less socio-economically disadvantaged [68,70,71], those who engaged in more physical activity [51], and in non-smokers [51,71]. Regardless, this cohort reported an average intake of just 1.3 servings (198 g/day) of fruit and 2.2 servings (168 g/day) of vegetables per day, consistent with previous findings of widespread inadequacy [3,16,22,27,52,58,71]. According to recommendations set out by the Australian Dietary Guidelines [40], these average intakes translated to only 22% of participants achieving recommendations for fruit (2 servings or 300 g), 1.5% achieving recommendations for vegetables (5–6 servings or 375–450 g), and only 0.7% achieving recommendations for both combined. This confirms the need for wide-reaching and novel methods to influence FVI across the entire Australian population. Nevertheless, targeting of messages is particularly warranted for smokers and sedentary individuals, each of which consistently reported both lower FVI and lower knowledge in all models, more so when considering the well-established harmful effects of smoking and sedentary behavior on CVD risk [72,73]. Although previous research in a subsample of this cohort [21] found that higher socio-economic status (SES) was positively associated with higher diet quality (measured by the Dietary Guidelines Index), we found little difference in FVI by either education or SEIFA disadvantage. In fact, those who were the most socio-economic disadvantaged actually reported higher fruit intake (9.6 g/day) when compared to those who were the least, although this was not evident for vegetables separately, nor for overall FVI combined. It appears that some studies show a positive association between SES and FVI [16,21,22], whilst others appear mixed [74,75], indicating further investigations are warranted. Briefly, we found those with CVD and/or diabetes had lower knowledge, and long-term FVI in these subgroups were less than adequate. Considering the positive impact of FVI on cardiometabolic disorders [7,8,9], targeting this subgroup is likely to attenuate disease progression and overall risk.

## 5. Strengths and Limitations

This study had several strengths. The data was part of Australia’s largest population-based diabetes study, representing both male and female adults across the lifespan. An extensive number of demographic and lifestyle variable were available to explore as potential confounders within our adjusted model, and FVI was assessed using a validated FFQ at three separate timepoints over 12 years. Furthermore, self-reported FVI has previously been validated against objectively measured serum carotenoids, a biomarker of FVI, in a subsample of this cohort [41]. The additional, albeit weak correlation between serum carotenoids and knowledge support results between FVK and FVI.

Some limitations were also present. Firstly, the cross-sectional nature of this study cannot assess causality. Secondly, we understand our knowledge questions reported only declarative (i.e., facts), not procedural (i.e., how to), knowledge. Thirdly, we recognize our knowledge questionnaire as a major limitation to the study, being a blunt tool that had not been formerly validated for the assessment of FVK, and, as such, this may have introduced the potential for bias. The field of nutrition knowledge only began to emerge in the early 2000s; therefore, at the time of data collection (1999/2000), validated knowledge instruments were not available [30]. The AusDiab FVK component of the questionnaire was limited to two simple questions, which were likely to capture only a component of an individual’s overall FVK and/or not fully assess a participants FVK; therefore, this may have influenced our results. The lack of homogeneity through the use of non-validated instruments to assess dietary knowledge appears to be a common limitation in similar studies [26,30]. Future research in this area is urgently needed to address this issue and always ensure well validated measures of FVK are available to accurately capture both declarative and procedural knowledge across the general population. However, objective biomarkers (i.e., serum carotenoids) have previously positively correlated with FVI [41], and then weakly, but significantly, with FVK in the current investigation, indicating a positive association between FVK and FVI. Furthermore, the interpretation and weighting applied to responses from the knowledge questionnaire to create our knowledge scores are recognized as a potential limitation. The nature of the questions asked, and the choice of responses offered [37], may have created an automated response of ‘daily’, thereby resulting in an inflation of participants in our ‘insufficient’ knowledge group. Whilst it could also be suggested that there was little difference in participants responding ‘daily’ to ‘several times a day’, it is very unlikely one can successfully reach the recommended FVI in a single setting of ‘daily’, therefore adding weight to our decision to assign ‘several times a day’ as adequate. In addition, knowledge was also only measured at baseline, and we cannot rule out additional knowledge gains over time through other means. Nor, can we rule out the impacts of other major public health campaigns upon knowledge within the Australian landscape over this timeframe. Furthermore, we reported a 42 to 60% drop-out from baseline to the 5- and 12-year timepoints, which may have introduced selection bias. Lastly, the original cohort was randomly selected to optimize inferences toward the wider Australian population, albeit with low representation of indigenous or rural populations. Although cross-sectional weighting was applied to minimize for age and gender selection bias, participants were over-represented by higher education and higher socio-economic subgroups [36]. This may have impacted the generalizability of our results to lower socio-economic subgroups and possibly our ability to observe differences in FVI across SEIFA groups.

## 6. Conclusions

Our study demonstrated that FVI was highly inadequate among this Australian adult cohort and that intake could be increased by improving fruit and vegetable knowledge. Public health messages should have greater focus on improving knowledge of fruit and vegetable recommendations to encourage higher FVI across all meals, particularly for vegetables. Furthermore, these messages likely require repeated exposures over time to reach and maintain their potential benefits for public health. In addition, providing targeted messages that highlight the need to increase FVI in subgroups for whom intake is particularly low, such as males, smokers, and for those that are sedentary or have low physical activity levels, is warranted. There are many complexities to changing dietary behavior; however, we have found that essentially stripping back to one important aspect of knowledge, i.e., knowing how often to consume fruit and vegetables for a well-balanced diet, is an important component of nutrition literacy and potentially increasing FVI. Yet, according to our study, only one-quarter of Australian adults acquire this knowledge. Whilst various theoretical frameworks can be used to influence dietary behavior, establishing FVK is an integral step in health promotion. To our knowledge, we are the first to show that raising awareness of the need to consume fruit and vegetables several times a day for a well-balanced diet could potentially improve medium to long-term FVI.

## Figures and Tables

**Table 1:** Baseline characteristics for all participants and by fruit and vegetable knowledge score category.

		Baseline Knowledge of Fruit and Vegetable Intake		
	Total Cohort	Adequate	Insufficient	Poor
*n* (%)	8966	2085 (24.1)	6596 (73.0)	285 (2.9)
Sex (men), *n* (%)	4043 (49.8)	563 (32.6)	3291 (54.8)	189 (67.5)
Age groups, *n* (%)				
25–45 years	3127 (47.8)	970 (60.1)	2060 (43.7)	97 (47.6)
45–65 years	4188 (35.7)	930 (31.3)	3144 (37.4)	114 (30.4)
>65 years	1651 (16.5)	185 (8.6)	1392 (18.9)	74 (22.0)
BMI groups, *n* (%)				
Underweight	85 (1.0)	19 (1.2)	64 (0.8)	2 (3.5)
Normal	3269 (39.4)	864 (43.6)	2324 (38.3)	81 (34.0)
Overweight	3621 (39.3)	721 (35.1)	2758 (40.6)	142 (41.5)
Obese	1991 (20.3)	481 (20.2)	1450 (20.3)	60 (21.0)
Energy intake (MJ/day), mean ± SD	8.8 ± 3.1	8.5 ± 2.9	8.8 ± 3.1	8.9 ± 3.9
Physical activity, *n* (%)				
Sedentary	1523 (15.8)	269 (11.8)	1183 (17.2)	71 (15.2)
Insufficient	2761 (32.1)	679 (33.7)	1997 (31.3)	85 (38.6)
Sufficient	4682 (52.1)	1137 (54.5)	3416 (51.5)	129 (46.2)
Relationship status, *n* (%)				
Married	6495 (72.6)	1532 (70.8)	4772 (73.5)	191 (65.6)
De facto	434 (4.5)	115 (5.4)	300 (4.3)	19 (4.4)
Separated	228 (2.4)	61 (3.9)	157 (1.8)	10 (4.7)
Divorced	534 (5.1)	119 (5.0)	396 (5.2)	19 (4.7)
Widowed	559 (5.3)	82 (3.4)	453 (5.8)	24 (8.5)
Single	716 (10.0)	176 (11.5)	518 (9.4)	22 (12.1)
Level of education, *n* (%)				
Never to some high school	3586 (35.9)	668 (29.8)	2773 (37.7)	145 (40.7)
Completed university/equivalent	5380 (64.1)	1417 (70.2)	3823 (62.3)	140 (59.3)
SEIFA Disadvantage, *n* (%)				
Quartile 1 (least disadvantaged)	2634 (35.1)	695 (38.1)	1889 (34.5)	50 (25.8)
Quartile 2	3501 (34.3)	860 (34.8)	2539 (34.3)	102 (32.0)
Quartile 3	1437 (16.1)	283 (16.0)	1088 (15.9)	66 (22.0)
Quartile 4 (most disadvantaged)	1394 (14.5)	247 (11.1)	1080 (15.4)	67 (20.3)
Smoking status, *n* (%)				
Current	1366 (15.7)	207 (9.7)	1091 (17.2)	68 (29.1)
Former smoker	2628 (25.8)	578 (25.0)	1951 (26.0)	99 (29.9)
Non-smoker	4972 (58.4)	1300 (65.3)	3554 (56.8)	118 (41.0)
Self-reported CVD history, Yes, *n* (%)	718 (6.8)	89 (3.9)	599 (7.5)	30 (12.4)
Diabetes status, *n* (%)				
Normal glucose levels	6620 (76.9)	1686 (83.8)	4745 (75.0)	189 (67.0)
Known Diabetes Mellitus	357 (3.4)	46 (2.3)	293 (3.6)	18 (6.3)
Impaired fasting glucose	534 (5.8)	85 (4.2)	423 (6.1)	26 (10.2)
Impaired glucose tolerance	1088 (10.4)	218 (8.0)	835 (11.2)	35 (11.9)
New Diabetes Mellitus	367 (3.5)	50 (1.7)	300 (4.1)	17 (4.6)

All *n* values are the number of observations, weighted using survey command. Baseline fruit and vegetable knowledge score: Poor defined as <40, Insufficient as 40–45, Adequate as 50; BMI body mass index; Underweight defined as <18.5 kg/m^2^, Normal as 18.5–24.9 kg/m^2^, Overweight as ≥25 kg/m^2^, and Obese as ≥30 kg/m^2^; Physical activity levels defined as Sedentary nil physical activity time, Insufficient as <150 min/week and Sufficient as >150 min/week; SEIFA Disadvantage scores defined as Quartile 1 as >1068 (least disadvantaged), Quartile 2 as 1068–990, Quartile 3 as 989–928, and Quartile 4 as <928 (most disadvantaged); MJ, megajoules; CVD, cardiovascular disease.

**Table 2:** Relationship of fruit and vegetable intake (combined and discretely) with knowledge of fruit and vegetable intake at baseline, *n* = 8966 ^a^.

	Baseline Knowledge of Fruit and Vegetable Intake		
	Adequate	Insufficient	Poor
FVI (grams/day)			
*n*, (%)	2085 (24.1)	6596 (73.0)	285 (2.9)
Unadjusted	reference	−57.5 (−73.4, −41.7)	−125.8 (−144.7, −106.9)
Multivariable adjusted ^b^	reference	−67.1 (-80.0, −54.3)	−124.0 (−142.9, −105.1)
Fruit Intake (grams/day)			
*n*, (%)	3754 (42.6)	5031 (55.6)	181 (1.8)
Unadjusted	reference	−65.8 (−74.2, −57.3)	−107.1 (−130.0, −84.1)
Multivariable adjusted ^b^	reference	−65.3 (−73.3, −57.3)	−96.7 (−126.9, −66.4)
Vegetable Intake (grams/day)			
*n*, (%)	2775 (32.2)	6032 (65.9)	159 (1.9)
Unadjusted	reference	−11.2 (−21.1, −1.3)	−51.9 (−68.1, −35.7)
Multivariable adjusted ^b^	reference	−16.1 (−26.4, −5.8)	−58.9 (−77.6, −40.2)

CI: confidence interval. ^a^ Results are presented as coefficient (95% CI) estimated using survey command for linear regression with the fruit and vegetable knowledge groups at baseline as the exposure of interest. The coefficient represents the difference (in grams/day) from the reference group. ^b^ Multivariable adjusted model included age, sex, BMI (body mass index), energy intake, relationship status, physical activity, level of education, SEIFA (Socio-economic index for areas) disadvantage, smoking status, diabetes status, and self-reported history of cardiovascular disease. Baseline fruit and vegetable knowledge score: Poor defined as <40, Insufficient 40–45, and Adequate as 50. Baseline fruit knowledge score: Poor defined as <20, Insufficient 20, and Adequate as 25. Baseline vegetable knowledge score: Poor defined as <20, Insufficient 20, and Adequate as 25.

**Table 3:** Relationship of the demographic and lifestyle factors in our adjusted model investigating the association of baseline fruit and vegetable knowledge scores with baseline fruit and vegetable intake.

	Baseline Fruit and Vegetable Intake (grams/day)		
Model 2 ^b^		Coefficient	(95% CI)
Baseline Knowledge Score	Adequate	reference	
	Insufficient	−67.1	(−80.0, −54.3)
	Poor	−124.0	(142.9, 105.1)
Sex	Male	reference	
	Female	25.7	(19.4, 32.0)
Age Groups	25–45 years	reference	
	45–65 years	59.3	(44.9, 73.6)
	>65 years	84.9	(64.4, 105.3)
BMI Groups	Normal	reference	
	Underweight	−36.6	(−92.2, 18.9)
	Overweight	1.6	(−10.5, 13.7)
	Obese	8.7	(−14.0, 31.4)
Energy Intake	(megajoules/day)	17.8	(16.0, 19.5)
Physical Activity Level	Sedentary	reference	
	Insufficient	2.5	(−17.6, 22.5)
	Sufficient	33.4	(19.4, 47.4)
Marital Status	Married	reference	
	De facto	−14.5	(−34.7, 5.7)
	Separated	−16.6	(−53.8, 20.5)
	Divorced	−16.1	(−42.2, 9.9)
	Widowed	−11.1	(−45.4, 23.2)
	Single	−2.3	(−20.8, 16.2)
Education Level	Never to some high school	reference	
	University or equivalent	−7.9	(−17.4, 1.6)
SEIFA Disadvantage	Quartile 1 (least)	reference	
	Quartile 2	0.1	(−16.3, 16.4)
	Quartile 3	8.3	(−15.4, 31.9)
	Quartile 4 (most)	10.9	(−19.2, 41.0)
Smoking Status	Current smoker	reference	
	Former smoker	59.1	(38.7, 79.5)
	Non-smoker	65.3	(45.1, 85.5)
Self-reported history of CVD	Yes	reference	
	No	−18.2	(−39.1, 2.7)
Diabetes Status	Normal glucose levels	reference	
	Known Diabetes Mellitus	35.6	(6.4, 64.7)
	New Diabetes Mellitus	−5.3	(−44.0, 33.5)
	Impaired fasting glucose	1.2	(−22.5, 24.9)
	Impaired glucose tolerance	−10.5	(−33.8, 12.8)

CI: confidence interval. ^b^ Results are presented as coefficient (95% CI) estimated using survey command for linear regression, adjusted for age group, sex, BMI, energy intake, relationship status, physical activity, level of education, SEIFA (Socio-economic index for areas) disadvantage, smoking status, diabetes status, and self-reported history of cardiovascular disease. Baseline fruit and vegetable knowledge score: Poor defined as <40, Insufficient 40–45, and Adequate as 50. Physical activity levels defined as Sedentary nil physical activity time, Insufficient as <150 min/week, and Sufficient as >150 min/week; SEIFA disadvantage scores defined as Quartile 1 as >1068 (least disadvantaged), Quartile 2 as 1068–990, Quartile 3 as 989–928, and Quartile 4 as <928 (most disadvantaged); BMI, body mass index; Underweight defined as <18.5 kg/m^2^, Normal as 18.5–24.9 kg/m^2^, Overweight as ≥25 kg/m^2^, and Obese as ≥30 kg/m^2^; CVD, cardiovascular disease.

**Table 4:** Relationship of total fruit and vegetable intakes at both 5 years (*n* = 5204) and 12 years (*n* = 3549), with knowledge of fruit and vegetable intake reported at baseline ^a^.

	Baseline Knowledge of Fruit and Vegetable Intake		
	Adequate	Insufficient	Poor
FVI (grams/day): 5 years			
*n*, (%)	1298 (24.9)	3775 (72.5)	131 (2.5)
Unadjusted	reference	−45.2 (−56.1, −34.3)	−128.2 (−159.3, −97.1)
Multivariable adjusted ^b^	reference	−50.4 (−61.4, −39.4)	−122.2 (−152.7, −91.6)
FVI (grams/day): 12 years			
*n*, (%)	1022 (28.8)	2457 (69.2)	70 (2.0)
Unadjusted	reference	−37.5 (−49.5, −25.6)	−92.0 (−131.7, −52.4)
Multivariable adjusted ^b^	reference	−42.5 (−54.6, −30.5)	−94.6 (−133.8, −55.5)

CI: confidence interval. ^a^ Results are presented as coefficient (95% CI) estimated using survey command for linear regression with the fruit and vegetable knowledge groups at baseline as the exposure of interest. The coefficient represents the difference (in grams/day) from the reference group. ^b^ Multivariable adjusted model included age, sex, BMI (body mass index), energy intake, relationship status, physical activity, level of education, SEIFA (Socio-economic index for areas) disadvantage, smoking status, diabetes status, and self-reported history of cardiovascular disease. Baseline fruit and vegetable knowledge score: Poor defined as <40, Insufficient 40–45, and Adequate as 50.

## Data Availability

Our researchers were granted access to data for this study from the AusDiab steering committee under a licensing agreement. Requests for its use and access for research are made possible through contact with the steering committee of AusDiab.

## Supplementary Materials

The following are available online at https://www.mdpi.com/2072-6643/12/12/3628/s1, Table S1: Baseline characteristics by category of fruit knowledge and vegetable knowledge, Table S2: Relationship of the demographic and lifestyle factors in our adjusted model investigating the association of baseline fruit knowledge and baseline vegetable knowledge scores with respective baseline fruit and vegetable intakes (grams/day).
