# Supplementary material for: Fruit and Vegetable Knowledge and Intake within an Australian Population: The AusDiab Study
Source: Nutrients. 2020 Nov 25;12(12):3628. doi: 10.3390/nu12123628 (PMC7759833; doi:10.3390/nu12123628)
Supplement: Supplementary file 1 [file nutrients-12-03628-s001.pdf]

nutrients

MDPI

Supplementary Materials

Table S1. Baseline characteristics by category of fruit knowledge and vegetable knowledge.

Characteristics	Baseline Knowledge of Fruit Intake			Baseline Knowledge of Vegetable Intake		
	**Adequate**	**Insufficient**	**Poor**	**Adequate**	**Insufficient**	**Poor**
n (%)	3,754 (42.6)	5,031 (55.6)	181 (1.8)	2,775 (32.2)	6,032 (65.9)	159 (1.9)
Sex (men), *n* (%)	1,277 (39.5)	2,652 (57.2)	114 (69.5)	832 (36.0)	3,102 (56.0)	109 (68.5)
Age groups, *n* (%)						
25-45 years	1,424 (50.4)	1,645 (45.6)	58 (51.2)	1,285 (59.6)	1,783 (41.9)	59 (50.8)
45-65 years	1,746 (34.4)	2,368 (37.0)	74 (28.4)	1,225 (31.6)	2,901 (38.0)	62 (26.9)
>65 years	584 (15.1)	1,018 (17.4)	49 (20.4)	265 (8.9)	1348 (20.1)	38 (22.2)
BMI groups						
Underweight	38 (1.1)	45 (0.7)	2 (5.8)	23 (1.3)	61 (0.8)	1 (0)
Normal	1,428 (40.9)	1,791 (38.4)	50 (34.4)	1,116 (42.2)	2,110 (38.3)	43 (31.0)
Overweight	1,413 (37.6)	2,115 (40.6)	93 (42.5)	990 (35.7)	2,555 (41.1)	76 (38.9)
Obese	875 (20.4)	1,080 (20.3)	36 (17.3)	646 (20.8)	1,306 (19.7)	39 (30.0)
Energy intake (MJ/day), mean ± SD	8.6 ± 3.0	8.9 ± 3.1	9.2 ± 4.0	8.6 ± 3.0	8.8 ± 3.1	9.1 ± 3.2
Physical activity, *n* (%)						
Sedentary	564 (14.2)	914 (17.2)	45 (12.7)	376 (12.2)	1,108 (17.3)	39 (24.0)
Insufficient	1,172 (33.4)	1,527 (30.7)	62 (42.4)	903 (33.1)	1,819 (31.6)	39 (30.6)
Sufficient	2,018 (52.4)	2,590 (52.1)	74 (44.9)	1,496 (54.7)	3,105 (51.1)	81 (45.4)
Relationship status, *n* (%)						
Married	2,734 (72.5)	3,644 (72.8)	117 (68.7)	2,054 (72.0)	4,329 (72.9)	112 (71.2)
De facto	180 (4.6)	242 (4.6)	12 (2.8)	153 (5.3)	272 (4.1)	9 (5.5)
Separated	95 (3.3)	127 (1.7)	6 (3.3)	75 (3.3)	147 (1.9)	6 (4.3)
Divorced	239 (5.4)	279 (4.9)	16 (6.7)	152 (5.0)	374 (5.3)	8 (2.3)
Widowed	212 (4.7)	333 (5.9)	14 (3.7)	105 (3.1)	440 (6.3)	14 (10.2)
Single	294 (9.6)	406 (10.2)	16 (14.9)	236 (11.3)	470 (9.5)	10 (6.4)
Level of education, *n* (%)						
Never to some high school	1,396 (32.9)	2,096 (38.0)	94 (42.3)	897 (30.3)	2606 (38.2)	83 (51.5)

Completed university or equivalent	2,358 (67.1)	2,935 (62.0)	87 (57.7)	1,878 (69.7)	3,426 (61.8)	76 (48.5)
SEIFA Disadvantage, *n* (%)						
Quartile 1 (least)	1,179 (36.5)	1,426 (34.4)	29 (24.1)	897 (36.7)	1,711 (34.7)	26 (22.6)
Quartile 2	1,492 (35.0)	1,950 (34.1)	59 (25.4)	1,166 (36.0)	2,275 (33.7)	60 (31.1)
Quartile 3	575 (16.1)	818 (15.9)	44 (20.5)	389 (15.8)	1,008 (15.9)	40 (26.6)
Quartile 4 (most)	508 (12.4)	837 (15.6)	49 (30.0)	323 (11.5)	1,038 (15.8)	33 (19.7)
Smoking status, *n* (%)						
Current	419 (11.3)	902 (18.6)	45 (31.6)	316 (11.7)	1,013 (17.3)	37 (31.4)
Former smoker	1,087 (26.6)	1,484 (25.2)	57 (26.2)	787 (25.6)	1,786 (25.8)	55 (32.4)
Non-smoker	2,248 (62.1)	2,645 (56.1)	79 (42.2)	1,672 (62.7)	3,233 (57.0)	67 (36.2)
Self-reported CVD history, Yes, *n* (%)	234 (5.8)	462 (7.4)	22 (9.2)	130 (4.0)	573 (7.8)	15 (16.0)
Diabetes status, *n* (%)						
Normal glucose levels	2860 (79.3)	3643 (75.5)	117 (64.2)	2209 (82.0)	4305 (74.6)	106 (71.8)
Known Diabetes Mellitus	130 (3.5)	213 (3.2)	14 (6.0)	77 (2.7)	272 (3.7)	8 (5.3)
Impaired fasting glucose	187 (4.5)	331 (6.7)	16 (9.8)	132 (5.0)	385 (6.1)	17 (9.2)
Impaired glucose tolerance	443 (9.9)	622 (10.6)	23 (17.0)	283 (8.6)	787 (11.4)	18 (9.3)
New Diabetes Mellitus	134 (2.8)	222 (4.1)	11 (3.1)	74 (1.8)	283 (4.3)	10 (4.5)

All *n* values are the number of observations, weighted using survey command. Baseline fruit knowledge and baseline vegetable knowledge scores: Poor defined as <20, Insufficient as 20, Adequate as 25; BMI body mass index; Underweight defined as <18.5 kg/m^2; Normal as 18.5–24.9 kg/m^2; Overweight as ≥25 kg/m^2 and Obese as ≥30 kg/m^2; Physical activity levels defined as Sedentary nil physical activity time, Insufficient as <150 minutes/week and Sufficient as >150 minutes/week; SEIFA Disadvantage scores defined as Quartile 1 as >1068 (least disadvantaged), Quartile 2 as 1068 – 990, Quartile 3 as 989 – 928 and Quartile 4 as <928 (most disadvantaged); MJ megajoules; CVD cardiovascular disease.

Table S2. Relationship of the demographic and lifestyle factors in our adjusted model investigating the association of baseline fruit knowledge and baseline vegetable knowledge scores with respective baseline fruit and vegetable intakes (grams/day).

**Characteristics**	**Baseline Fruit Intake (grams/day)**		**Baseline Vegetable Intake (grams/day)**	
**Model 2[b]**	**Coefficient**	**(95% CI)**	**Coefficient**	**(95% CI)**
Baseline Knowledge Score				
Adequate	reference		reference	
Insufficient	–65.3	(–73.3, -57.3)	–16.1	(–26.4, -5.8)
Poor	–96.7	(–126.9, -66.4)	–58.9	(–77.6, -40.2)
Sex				
Male	reference		reference	
Female	13.9	(5.8, 21.9)	8.0	(0.7, 15.3)
Age Groups				
25–45 years	reference		reference	
45–65 years	40.4	(26.7, 54.1)	17.1	(8.5, 25.8)
>65 years	53.0	(33.5, 72.4)	27.1	(17.2, 37.1)
BMI Groups				
Normal	reference		reference	
Underweight	–37.9	(–76.8, 1.0)	-1.5	(–32.2, 29.1)
Overweight	–3.3	(–10.8, 4.3)	4.3	(–4.0, 12.6)
Obese	0.9	(–15.6, 17.3)	7.8	(–1.4, 16.9)
Energy Intake (MJ/day)	8.5	(7.2, 9.8)	9.1	(7.8, 10.3)
Physical Activity Level				
Sedentary	reference		reference	
Insufficient	0.0	(–18.7, 18.8)	1.5	(–6.5, 9.5)
Sufficient	24.2	(11.7, 36.8)	9.0	(1.4, 16.6)
Marital Status				
Married	reference		reference	
De facto	–19.9	(–37.3, -2.5)	6.5	(–8.0, 20.9)
Separated	–18.2	(–45.5, 9.1)	–1.1	(–18.3, 16.1)
Divorced	–18.0	(–39.5, 3.4)	0.4	(–11.8, 12.7)
Widowed	5.0	(–24.0, 34.1)	–13.6	(–23.4, -3.7)

Single	3.0		–3.8	(–16.3, 8.6)
Education Level				
Never to some high school	reference		reference	
University or equivalent	2.1	(–5.4, 9.6)	-11.9	(–17.5, -6.2)
SEIFA Disadvantage				
Quartile 1 (least)	reference		reference	
Quartile 2	–4.4	(–21.0, 12.1)	3.5	(–7.5, 14.4)
Quartile 3	–1.7	(–22.2, 18.9)	9.7	(–11.5, 30.9)
Quartile 4 (most)	9.6	(0.1, 19.2)	1.9	(–28.0, 31.8)
Smoking Status				
Current smoker	reference		reference	
Former smoker	43.9	(28.0, 59.7)	12.2	(0.5, 23.8)
Non-smoker	53.2	(35.9, 70.5)	10.2	(0.0, 20.3)
Self-reported history of CVD				
Yes	reference		reference	
No	–6.3	(–25.7, 13.1)	–12.9	(–19.5, -6.2)
Diabetes Status				
Normal glucose levels	reference		reference	
Known Diabetes Mellitus	24.4	(–5.8, 54.6)	6.5	(–5.8, 18.7)
New Diabetes Mellitus	3.6	(–24.9, 32.2)	–8.7	(–24.5, 7.0)
Impaired fasting glucose	5.0	(–10.6, 20.6)	–1.9	(–16.3, 12.5)
Impaired glucose tolerance	–8.7	(–23.8, 6.4)	–2.3	(–14.0, 9.4)

CI: confidence interval. [b] Results are presented as coefficient (95% CI) estimated using survey command for linear regression, adjusted for age, sex, BMI, energy intake, relationship status, physical activity, level of education, SEIFA (Socio-economic index for areas) disadvantage, smoking status, diabetes status and self-reported history of cardiovascular disease. Baseline fruit knowledge score: Poor defined as <20, Insufficient 20, and Adequate as 25. Baseline vegetable knowledge score: Poor defined as <20, Insufficient 20, and Adequate as 25. Physical activity levels defined as: Sedentary nil physical activity time, Insufficient as <150 minutes/week and Sufficient as >150 minutes/week; SEIFA disadvantage scores defined as Quartile 1 as >1068 (least disadvantaged), Quartile 2 as 1068 – 990, Quartile 3 as 989 – 928, and Quartile 4 as <928 (most disadvantaged); BMI body mass index; Underweight defined as <18.5 kg/m^2, Normal as 18.5-24.9 kg/m^2, Overweight as ≥25 kg/m^2 and Obese as ≥30 kg/m^2; MJ megajoules; CVD cardiovascular disease.
